# New Phenylethanoid Glycosides from the Fruits of *Forsythia Suspense* (Thunb.) Vahl

**DOI:** 10.3390/molecules14031324

**Published:** 2009-03-25

**Authors:** Fu Nan Wang, Zhi Qiang Ma, Ying Liu, Ying Zhi Guo, Zhong Wei Gu

**Affiliations:** 1 Graduate School, Peking Union Medical College, Beijing 100005, P.R.China; E-mail: fnwang9966@yahoo.com.cn (F-N.W.); 2 National Research Institute for Family Planning, Beijing 100081, P.R.China; 3 Harbin Pharm. Group Co., Ltd., Second Chinese Medicine Factory, Harbin,150078, P.R.China; E-mail: mazhiqiangsapo@tom.com (Z-Q.M.); 4 Institute of Materia Medica, Chinese Academy of Medical Sciences and Peking Union Medical College, Beijing 100050, China; E-mail: liuying@imm.ac.cn (Y. L.); 5 National Engineering Research Center for Biomaterials, Sichuan University, Chengdu 610064, P.R.China

**Keywords:** Caffeoyl phenylethanoid glycosides, *Forsythia suspense* (Thunb.) Vahl., Forsythosides H-J.

## Abstract

Forsythosides H-J (**1-3**), three new caffeoyl phenylethanoid glycosides (CPGs), were isolated from the fruits of *Forsythia suspense* (Thunb.) Vahl., together with six known phenylethanoid glycosides: Forsythoside A (**4**), Forsythoside F (**5**), Forsythoside E (**6**), 2-(3,4-dihydroxyphenyl)ethyl-*β*-d-glucopyranoside (**7**), phenethyl alcohol *β*-d-xylo-pyranosyl-(1→6)-*β*-d-glucopyranoside (**8**) and calceolarioside B (**9**). Their structures were determined by spectroscopic and chemical methods.

## 1. Introduction

*Forsythia suspense* (Thunb.) Vahl. is widely distributed in China, Korea and Japan. The fruits of this plant, known as “Lianqiao” (Chinese), have been used as a Chinese traditional medicine to treat inflammation, pyrexia, ulcer, gonorrhea and erysipelas [1]. A number of chemical constituents with diverse structures, including phenylethanoid glycosides [2,3,4,5,6,7,8,9,10,11], lignans [12,13,14] and flavonoids [2,15] have been reported from species of this genus. The interesting chemical, pharmacological, and clinical significance of *Forsythia suspense* (Thunb.) Vahl. prompted us to carry out the current project, which has led to the isolation of three new caffeoyl phenylethanoid glycosides **1**-**3** and six known compounds.

## 2. Results and Discussion

**Figure 1:**
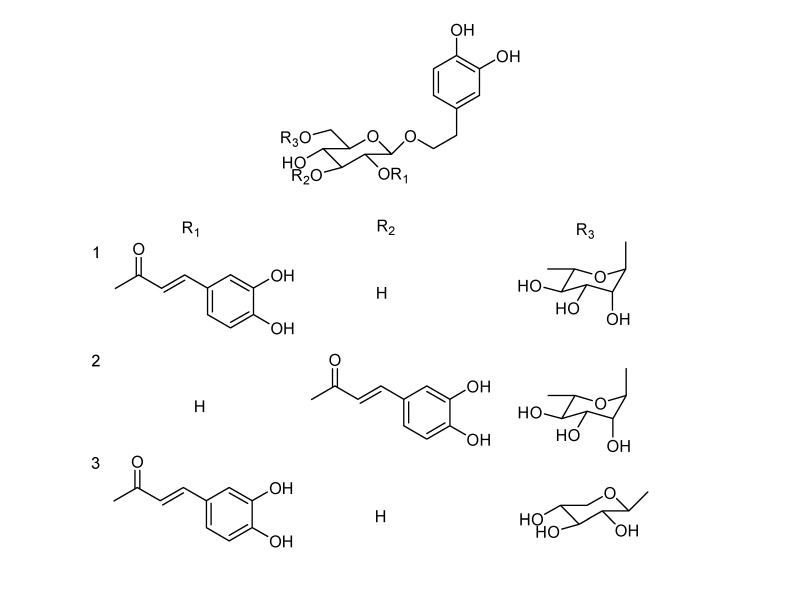
Structures of Forsythoside H (**1**), Forsythoside I (**2**) and Forsythoside J (**3**).

Repeated column chromatography of the extract of *Forsythia suspense* (Thunb.) Vahl. yielded three new caffeoyl phenylethanoid glycosides designated as Forsythosides H-J (**1**-**3**, Figure 1), together with six known phenylethanoid glycosides. These known compounds were identified as Forsythoside A (**4**) [10], Forsythoside F (**5**) [12], Forsythoside E (**6**) [6], 2-(3,4-dihydroxyphenyl)ethyl*β*-d-gluco-pyranoside (**7**) [17], phenethyl alcohol *β*-d-xylopyranosyl-(1→6)-*β*-d-glucopyranoside (**8**) [18] and calceolarioside B (**9**) [19] by comparison of their spectroscopic data (UV, IR, ESIMS, ^1^H- and ^13^C-NMR) with that reported in the literature.

Forsythoside H (**1**) was obtained as a brown amorphous powder. The presence of hydroxyl (3,339 cm^-1^) and carbonyl (1,694 cm^-1^) groups were evident in its IR spectrum. The negative mode ESIMS of **1** gave a quasi-molecular ion peak at *m/z* 623 [M-H]^-^. Its molecular formula, C_29_H_36_O_15_, was established by HRESIMS 623.1960 (calcd. for C_2__9_H_3__5_O_1__5_: 623.1976), corresponding to twelve degrees of unsaturation. The ^1^H-NMR spectrum revealed the presence of two sets of ABX systems [*δ* 6.54 (br.s), *δ* 6.55 (d, *J* = 8.4 Hz) and *δ* 6.41 (dd, *J* = 8.4, 1.8Hz) for the 3,4-dihydroxyphenylethyl moiety; and *δ* 7.06 (br.s), *δ* 6.76 (d, *J* = 7.2 Hz) and *δ* 7.01 (br.d, *J* = 7.2 Hz) for the caffeoyl moiety], two trans-olefinic protons [*δ* 6.27 and 7.49 (each d, *J* = 16.2 Hz)], together with two anomeric protons at *δ* 4.49 (d, *J* = 8.4 Hz) for *β*-glucose, and *δ* 4.60 (br.s) for *α*-rhamnose.

**Table 1:** NMR Data for Compounds **1**-**4**^ a^.

no.	**1**		**2**		**3**		**4**	
	*δ*_H_	*δ*_C_	*δ*_H_	*δ*_C_	*δ*_H_	*δ*_C_	*δ*_H_	*δ*_C_
1		129.1		129.5		129.2		129.2
2	6.54 br.s	115.4	6.61 d (1.8)	115.5	6.55 d (1.2)	115.4	6.62 br.s	115.5
3		144.9		144.7		144.9		144.9
4		143.5		143.5		143.5		143.5
5	6.55 d (8.4)	116.2	6.63 d (7.8)	116.3	6.54 d (7.8)	116.2	6.63 d (7.8)	116.3
6	6.41 dd (1.8, 8.4)	119.6	6.48 dd (1.8, 7.8)	119.5	6.41 dd (1.2, 7.8)	119.6	6.49 dd (1.8, 7.8)	119.5
7	2.56 m	35.1	2.69 m	35.1	2.56 m	35.0	2.67 m	35.1
8	3.76 m	69.8	3.82 m	70.2	3.78 m	69.8	3.83 m	70.3
	3.54 m		3.62 m		3.53 m		3.61 m	
1′	4.49 d (8.4)	100.2	4.34 d (7.8)	102.7	4.74 d (7.8)	100.1	4.31 d (7.8)	102.9
2′	4.64 t (8.4)	73.4	3.18 dd (7.8, 9.0)	71.4	4.65 t (7.8)	73.3	3.41 dd (7.8, 9.0)	73.0
3′	3.42 m	74.1	4.88 t (9.0)	77.5	3.42 m	74.1	3.10 m	73.5
4′	3.44 m	70.7	3.28 dd (9.0, 10.2)	68.1	3.22 dd (9.0, 9.6)	70.0	4.66 t (9.6)	71.0
5′	3.38 m	75.5	3.42 m	75.1	3.39 m	75.7	3.45 m	73.9
6′	3.84 br.d (10.2)	66.6	3.82 br.d (9.6)	66.5	3.96 br.d (11.4)	65.7	3.53 br.d (13.0)	66.1
	3.48 m		3.49 m		3.58 dd (5.4, 11.4)		3.33 dd (7.5, 13.0)	
1″	4.60 br.s	100.7	4.59 br.s	100.7	4.20 d (7.2)	104.0	4.50 br.s	100.5
2″	3.63 m	70.5	3.62 m	70.6	2.98 dd (7.2, 8.4)	73.3	3.59 m	70.6
3″	3.45 m	70.3	3.62 m	70.4	3.09 dd (8.4, 9.0)	76.6	3.36 dd (9.0, 10.2)	70.6
4″	3.19 t (9.6)	72.0	3.44 m	71.9	3.28 m	69.6	3.57 t (9.0)	71.9
5″	3.46 m	68.4	3.48 m	68.4	3.70 m	68.2	3.34 m	68.4
					3.02 m			
6″	1.14 d (6.6)	18.0	1.13 d (6.6)	17.9			1.05 d (6.0)	17.8
1‴		125.5		125.6		125.5		125.4
2‴	7.06 br.s	114.8	7.04 d (1.8)	114.9	7.06 br.s	114.8	7.05 br.s	114.8
3‴		145.6		145.5		145.6		145.6
4‴		148.5		148.2		148.5		148.6
5‴	6.76 d (7.2)	115.8	6.75 d (8.4)	115.7	6.76 d (7.8)	115.8	6.76 d (8.4)	115.8
6‴	7.01 br.d (7.2)	121.3	7.01 dd (1.8, 8.4)	121.3	7.01 d (7.8)	121.3	7.01 br.d (8.4)	121.4
7‴	7.49 d (16.2)	145.2	7.47 d (15.6)	144.9	7.49 d (15.6)	145.1	7.50 d (15.6)	145.6
8‴	6.27 d (16.2)	114.2	6.26 d (15.6)	114.7	6.27 d (15.6)	114.2	6.25 d (15.6)	113.6
9‴		165.7		166.1		165.7		165.9

^a^ NMR data were measured in DMSO-*d*_6_ for **1**-**4** at 400 MHz for ^1^H-NMR and at 100 MHz for ^13^C-NMR. Proton coupling constants (*J*) in Hz are given in parentheses. The assignments were based on DEPT, ^1^H-^1^H COSY, HSQC, HMBC and phase sensitive ^1^H-^1^H COSY experiments.

**Figure 2:**
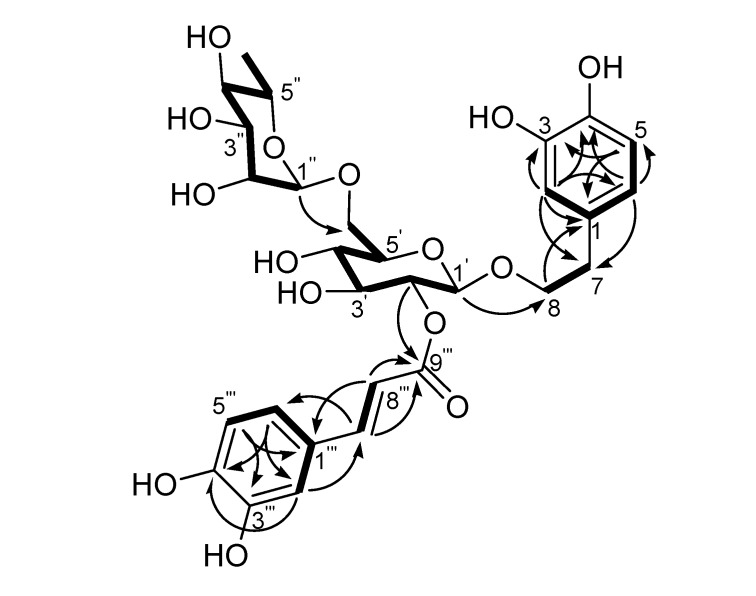
Main ^1^H-^1^H COSY (thick lines) and HMBC (arrows from proton to carbon) correlations of Forsythoside H (**1**).

Acid hydrolysis of **1** yielded d-glucose and l-rhamnose in a ratio of 1:1 according to GC analysis of the trimethylsilyl-l-cysteine derivatives of the component monosaccharides, compared with the trimethylsilyl-l-cysteine derivatives of sugar standards. The NMR spectra of **1** were similar to those of the co-occurring Forsythoside A (**4**), with the only difference being in the position of the caffeoyl ester units, i.e. **1** is a positional isomer of **4**. Comparison of the ^13^C-NMR spectral data of **1** with those of **4**, showed the chemical shifts of C-1′, C-2′ and C-3′ were changed by -2.7, +0.4 and +0.6 ppm, respectively (Table 1), indicating that the caffeoyl residue is located at C-2. The ^1^H-NMR spectrum was in agreement with this, in particular, the low field position of H-2′ of the glucopyranosyl group (*δ*4.64) showed that this was the point of acylation. Analysis of the HMQC and ^1^H-^1^H COSY spectra of **1** led to the unambiguous assignment of proton and carbon signals in the NMR spectra. In the HMBC spectrum, two- and three-bond correlations (Figure 2, arrows) from H-1′ to C-8 and from H-1″ to C-6′, together with chemical shift values of these protons and carbons, revealed the connection among the 3,4-dihydroxyphenylethyl and the two sugar moieties of **1** was identical to that of Forsythoside A (**4**). Meanwhile, the location of the caffeoyl unit in **1** was indicated unequivocally by HMBC correlation from H-2′ to C-9‴. Accordingly, the structure of **1** was determined as 2-(3,4-dihydroxyphenyl)-ethyl-*O*-*α*-l-rhamnopyranosyl-(1→6)-2-*O*-trans-caffeoyl-*β*-d-glucopyranoside, and was named Forsythoside H.

Forsythoside I (**2**) was obtained as a brown amorphous powder, and its spectroscopic data (Table 1 and Experimental Section) indicated that it is another isomer of Forsythoside A (**4**)with a different connectivity between the caffeoyl and glucopyranosy moieties. Acid hydrolysis of **2** released d-glucose and l-rhamnose, identified by GC analysis. In the ^13^C-NMR spectrum of **2**, a characteristic resonance at *δ*_C_ 77.5 ppm indicated a (9‴→3′) connection between the caffeoyl moiety and glucopyranosy moiety [8]. The NMR data assignments (Table 1) and structure of **2** were established by 2D NMR experiments. Thus, compound **2** was determined to be 2-(3,4-dihydroxyphenyl)-ethyl-*O*-*α*-l-rhamnopyranosyl-(1→6)-3-*O*-trans-caffeoyl-*β*-d-glucopyranoside, named as Forsythoside I.

Forsythoside J (**3**) was obtained as a brown amorphous powder. It showed a quasi-molecular ion peak at *m/z* 609 [M-H]^-^. The ^1^H- and ^13^C-NMR spectroscopic data indicated that compound **3** is a caffeoyl phenylethanoid glycoside with two sugar moieties. The chemical shifts of compound **3** were almost the same as those of **1**. However, a pair of proton signals attributed to a *β*-xylopyranosyl unit replaced those of the outer *α*-rhamnopyranosyl of **1**. Acid hydrolysis of **3** produced d-glucose and d-xylose in a ratio of 1:1 by GC analysis of the trimethylsilyl-l-cysteine derivatives of the component monosaccharides. These data demonstrated that **3** is an analogue of **1** with an outer *β*-xylopyranosyl unit. Unambiguous assignments of the NMR data of **3** (Table 1) were accomplished from the 2D NMR spectra. In the HMBC spectrum long-range correlations of H-1″ to C-6′ indicated that the *β*-xylo-pyranosyl moiety of **3** was located at C-6′. Therefore, **3** was established as 2-(3,4-dihydroxyphenyl)-ethyl-*O*-*β*-d-xylopyranosyl-(1→6)-2-*O*-trans-caffeoyl-*β*-d-glucopyranoside, and it was named Forsythoside J.

## 3. Experimental

### 3.1. General

IR spectra were recorded as KBr disks on Shimadzu FTIR-8700 (Shimadzu Co. Japan). 1D and 2d-NMR spectra were obtained at 400 MHz for ^1^H and at 100 MHz for ^13^C, respectively, on a Bruker AV400 spectrometer in DMSO-*d*_6_ with TMS as references. ESIMS data were measured with a Q-Trap LC/MS/MS (Turbo Ionspray source) spectrometer. HRESIMS data were measured on an AccuToFCS JMS-T100CS spectrometer. GC data were measured on a Perkin Elmer Autosystem XL Gas Chromatograph instrument. Column chromatography was performed with silica gel (200–300 mesh, Qingdao Marine Chemical Inc., Qingdao, People’s Republic of China) and Sephadex LH-20 (Pharmacia Biotech AB, Uppsala, Sweden). Preparative HPLC separation (Agilent 1100) was carried out on a reversed-phase column using a differential refractometer detector. TLC was carried out with glass precoated silica gel GF_254_ plates. Spots were visualized under UV light or by spraying with 5% H_2_SO_4_ in 95% EtOH, followed by heating. 

### 3.2. Plant Material

The fruits of *Forsythia suspense* (Thunb.) Vahl. were collected at Shanxi Province, People’s Republic of China, in September 2006. The plant identification was verified by Professor Qi-shi Sun (Shenyang Pharmaceutical University). A voucher specimen was deposited in the Herbarium of Shool of Traditional Chinese Medicines of Shenyang Pharmaceutical University, China.

### 3.3. Extraction and Isolation.

The fruits of *Forsythia suspense* (Thunb.) Vahl. (4.0 kg) were extracted with 85% EtOH under reflux. After concentration *in vacuo*, the crude EtOH extract (1.8 kg) was suspended in water and partitioned successively with petroleum ether, ethyl acetate (EtOAc), and *n*-butanol. The *n*-butanol-soluble part (182.5 g) was subjected to normal silica gel column chromatography, eluting with a gradient of increasing MeOH (0-50%) in CHCl_3_, afford seven fractions A-G. Fraction C (36.2 g) was subjected to column chromatography, using CHCl_3_-MeOH-H_2_O as the eluting solvent, to afford six subfractions C_1_-C_6_. Subfraction C_2_ (164.3 mg) and C_3_ (150.6 mg) were separately purified by reversed-phase preparative HPLC, using MeOH-H_2_O (25:75 and 30:70) as the mobile phases, respectively, to afford **1** (9.5 mg), **2** (10.2 mg), **3** (12.3 mg), and **4** (16.1 mg) and **5** (13.6 mg). Subfraction C_6_ (185.6 mg) was further separated by silica gel column chromatography, using EtOAc-MeOH-H_2_O as the eluting solvent, and then purified by reversed-phase preparative HPLC, using MeOH-H_2_O (45:65) as the mobile phases, to yield **6** (14.2 mg), **7** (14.2 mg), **8** (8.9 mg) and **9** (32.5 mg).

*Forsythoside H* (**1**): a brown amorphous powder; IR (KBr) ν_max_ cm^-1^: 3,339, 2,941, 1,694, 1,601, 1,518; ^1^H-NMR and ^13^C-NMR data, see Table 1; HRESIMS *m/z* 623.1960 (calcd. for C_2__9_H_3__5_O_1__5_: 623.1976).

*Forsythoside* *I* (**2**): a brown amorphous powder; IR (KBr) ν_max_ cm^-1^: 3,361, 2,933, 1,692, 1,601, 1,519; ^1^H-NMR and ^13^C-NMR data, see Table 1; HRESIMS *m/z* 623.1962 (calcd. for C_2__9_H_3__5_O_1__5_: 623.1976).

*Forsythoside* *J* (**3**): a brown amorphous powder; IR (KBr) ν_max_ cm^-1^: 3,340, 2,933, 1,693, 1,597, 1,499; ^1^H-NMR and ^13^C-NMR data, see Table 1; HRESIMS *m/z* 609.1813 (calcd, for C_2__8_H_3__3_O_1__5_: 609.1813).

### 3.4. Acid Hydrolysis of ***1***, ***2*** and ***3***

Each glycoside (5 mg) was refluxed in 2 N HCl for 3 h at 80 °C. The reaction mixture was extracted with CHCl_3_ (3 × 5 mL) and the aqueous phase was neutralized with 1 N NaOH and dried using a stream of N_2_. The residue were separately subjected to CC over silica gel with MeCN-H_2_O (9:1) as the eluent to yield d-glucose and l-rhamnose from **1** and **2**, and d-glucose and d-xylose from **3**, respectively [20]. The sugar residue was then dissolved in pyridine (1 mL) and l-cysteine methyl ester hydrochloride (2 mg) was added. The mixture was left at 60 °C for 2 h and evaporated under a N_2_ stream and dried *in* *vacuo*. The residue was trimethylsilylated with *N-*trimethylsilylimidazole (0.2 mL) at 60 °C for 1 h. The mixture was partitioned between *n*-hexane and H_2_O (3 × 1 mL), and the *n*-hexane extract was subjected to GC analysis to identify the sugars. Capillary column DB-5 (30 m × 0.25 mm × 0.25 *μ*m); detection FID; detector temperature 280 °C; injection temperature 250 °C; the initial column temperature was 100 °C, and the temperature was gradually raised to 280 °C at the rate of 10 °C/min and maintained for 5 min; carrier N_2_ gas. Retention times for d-glucose, d-xylose, and l-rhamnose were 19.6, 17.6, and 18.4 min, respectively.

## References

[1] (2005). State Pharmacopeia Commission of P. R. China. Pharmacopeia of the P. R. China.

[2] Tokar M., Klimek B. (2004). Isolation and identification of biologically active compounds from Forsythia viridissima flowers. Acta Pol. Pharm..

[3] Nishibe S., Okabe K., Tsukamoto H., Sakushima A., Hisada S. (1982). Studies on the Chinese crude drug Forsythia fructus. V. The structure of forsythiaside isolated from *Forsythia suspensa*. Chem. Pharm. Bull..

[4] Ming D.-S, Yu D.-Q., Yu S.-S. (1999). Two new caffeoyl glycosides from Forsythia suspensa. J. Asian Nat. Prod. Res..

[5] Nishibe S., Okabe K., Tsukamoto H., Sakushima A., Hisada S., Baba H., Akisada T. (1982). Studies on the Chinese crude drug “forsythiae fructus”. VI. The structure and antibacterial activity of suspensaside isolated from Forsythia suspensa. Chem. Pharm. Bull..

[6] Endo K., Hikino H. (1984). Structures of rengyol, rengyoxide, and rengyolone, new cyclohexylethane derivatives from Forsythia suspensa fruits. Can. J. Chem..

[7] Kitagawa S., Tsukamoto H., Hisada S., Nishibe S. (1984). Studies on the Chinese crude drug “forsythiae fructus”. VII. A new caffeoyl glycoside from Forsythia viridissima. Chem. Pharm. Bull..

[8] Liu D.-L., Zhang Y., Xu S.-X., Xu Y., Wang Z.-X. (1998). Phenylethanoid glycosides from Forsythia suspensa Vahl. J. Chin. Pharm. Sci..

[9] Endo K., Hikino H. (1982). Validity of oriental medicine. Part 44. Structures of forsythoside C and D, antibacterial principles of Forsythia suspensa fruits. Heterocycles.

[10] Endo K., Takahashi K., Abe T., Hikino H. (1981). Structure of forsythoside A, an antibacterial principle of Forsythia suspensa leaves. Heterocycles.

[11] Endo K., Takahashi K., Abe T., Hikino H. (1982). Structure of forsythoside B, an antibacterial principle of Forsythia koreana stems. Heterocycles.

[12] Endo K., Takahashi K. (1990). Constitutions of forsythosides F and G, new phenol glycosides of Forsythia viridissima stems. Heterocycles.

[13] Rahman M. M. A., Dewick P. M., Jackson D. E., Lucas J. A. (1990). Lignans of Forsythia intermedia. Phytochemistry.

[14] Liu D.-L., Xu S.-X., Wang W.-F. (1998). A novel lignan glucoside from Forsythia suspensa Vahl. J. Chin. Pharm. Sci..

[15] Kitagawa S., Nishibe S., Benecke R., Thieme H. (1988). Phenolic compounds from Forsythia leaves. II. Chem. Pharm. Bull..

[16] Matsuo K., Tokoroyama T., Kubota T. (1972). Bitter constituents of Forsythia viridissima. Phytochemistry.

[17] Lee  T. H., Kuo Y. C., Wang G. J., Kuo Y. H., Chang  C. I., Lu  C. K., Lee  C. K. (2002). Five new phenolics from the roots of Ficus beecheyana. J. Nat. Prod..

[18] Otsuka H., Takeda Y., Yamasaki K. (1990). Xyloglucosides of benzyl and phenethyl alcohols and Z-hex-3-en-1-ol from leaves of Alangium platanifolium var. trilobum. Phytochemistry.

[19] Damtoft  S., Jensen S. R. (1994). Three phenylethanoid glucosides of unusual structure from *Chirita sinensis* (gesneriaceae). Phytochemistry.

[20] Kinjo J., Araki K., Fukui K., Higuchi  H., Ikeda  T., Nohara T., Ida  Y., Takemoto  N., Miyakoshi  M., Shoji  J. (1992). Six new triterpenoidal glycosides including two new sapogenols from Albizziae Cortex. V. Chem. Pharm. Bull..

